# Synergistic effect of *imp/ostA *and *msbA *in hydrophobic drug resistance of *Helicobacter pylori*

**DOI:** 10.1186/1471-2180-9-136

**Published:** 2009-07-13

**Authors:** Hung-Chuan Chiu, Tzu-Lung Lin, Jyh-Chin Yang, Jin-Town Wang

**Affiliations:** 1 Department of Microbiology, National Taiwan University College of Medicine; Taipei City 10051, Taiwan, Republic of China; 2 Department of Internal Medicine, National Taiwan University Hospital, Taipei City 10002, Taiwan, Republic of China

## Abstract

**Background:**

Contamination of endoscopy equipment by *Helicobacter pylori *(*H. pylori*) frequently occurs after endoscopic examination of *H. pylori*-infected patients. In the hospital, manual pre-cleaning and soaking in glutaraldehyde is an important process to disinfect endoscopes. However, this might not be sufficient to remove *H. pylori *completely, and some glutaraldehyde-resistant bacteria might survive and be passed to the next patient undergoing endoscopic examination through unidentified mechanisms. We identified an Imp/OstA protein associated with glutaraldehyde resistance in a clinical strain, NTUH-C1, from our previous study. To better understand and manage the problem of glutaraldehyde resistance, we further investigated its mechanism.

**Results:**

The minimal inhibitory concentrations (MICs) of glutaraldehyde andexpression of *imp/ostA *RNA in 11 clinical isolates from the National Taiwan University Hospital were determined. After glutaraldehyde treatment, RNA expression in the strains with the MICs of 4–10 μg/ml was higher than that in strains with the MICs of 1–3 μg/ml. We examined the full-genome expression of strain NTUH-S1 after glutaraldehyde treatment using a microarray and found that 40 genes were upregulated and 31 genes were downregulated. Among the upregulated genes, *imp/ostA *and *msbA*, two putative lipopolysaccharide biogenesis genes, were selected for further characterization. The sensitivity to glutaraldehyde or hydrophobic drugs increased in both of *imp/ostA *and *msbA *single mutants. The *imp/ostA *and *msbA *double mutant was also hypersensitive to these chemicals. The lipopolysaccharide contents decreased in individual *imp/ostA *and *msbA *mutants and dramatically reduced in the *imp/ostA *and *msbA *double mutant. Outer membrane permeability assay demonstrated that the *imp/ostA *and *msbA *double mutation resulted in the increase of outer membrane permeability. Ethidium bromide accumulation assay demonstrated that MsbA was involved in efflux of hydrophobic drugs.

**Conclusion:**

The expression levels of *imp/ostA *and *msbA *were correlated with glutaraldehyde resistance in clinical isolates after glutaraldehyde treatment. Imp/OstA and MsbA play a synergistic role in hydrophobic drugs resistance and lipopolysaccharide biogenesis in *H. pylori*.

## Background

*Helicobacter pylori *was first isolated from the gastric mucosa of a patient with gastritis and peptic ulceration by Marshall and Warren in 1982 [1]. It is an important human pathogen, responsible for type B gastritis and peptic ulcers. Furthermore, infection by *H. pylori *is a risk factor for gastric adenocarcinoma and for lymphoma in the mucosa-associated lymphoid tissue of the stomach in humans [2-5].

*H. pylori *is believed to be transmitted from person to person by oral-oral or oral-fecal routes [6]. However, another possible route involves transmission during endoscopic examination of patients because contamination of endoscopy equipment by *H. pylori *frequently occurs after endoscopic examination of *H. pylori*-infected patients [7-9]. Because *H. pylori *is prevalent in the population [10], it is important to prevent its transmission. In the hospital, manual pre-cleaning and soaking in glutaraldehyde is an important process used to disinfect endoscopes [7,11]. However, endoscopic disinfection might not be sufficient to remove *H. pylori *completely [12,13]. Some glutaraldehyde-resistant bacteria might survive and be passed to the next person undergoing endoscopic examination through unidentified mechanisms. Therefore, it is an important issue to clarify the mechanism of glutaraldehyde resistance.

In our previous study, we demonstrated that the Imp/OstA protein was associated with glutaraldehyde resistance in a clinical strain of *H. pylori *[14]. *OstA *(*o*rganic *s*olvent *t*olerance) [15] has also been called *imp *(*i*ncreased *m*embrane *p*ermeability) [16], and was recently named *lptD *in *Escherichia coli *[17]. Imp/OstA exists widely in Gram-negative bacteria and participates in biogenesis of the cell envelope. It is an essential outer membrane protein in *E. coli*, depletion mutation of *imp/ostA *results in the formation of aberrant membranes [18]. Furthermore, Imp/OstA forms a complex with the RlpB lipoprotein and is responsible for lipopolysaccharide (LPS) assembly at the surface of the cell [17,19]. In addition, it mediates the transport of LPS to the surface in *Neisseria meningitidis *[20].

To further investigate the mechanism of glutaraldehyde resistance, we monitored the minimum inhibitory concentrations (MICs) and the expression of *imp/ostA *and Imp/OstA protein after glutaraldehyde treatment in 11 clinical isolates. Full-genome expression was also studied by microarray analysis; 40 genes were upregulated and 31 genes were downregulated in NTUH-S1 after glutaraldehyde treatment. Among the upregulated genes, *msbA*, was selected for further study. MsbA is an essential inner membrane protein in *E. coli *and a member of the ABC transporter superfamily of proteins [21]. MsbA produced in the Gram-positive organism *Lactococcus lactis *is capable of conferring drug resistance to the organism [22]. In addition, *msbA *is not essential in *N. meningitidis *and this organism can survive without LPS [23]. In *E. coli*, *msbA *was implicated in lipid A-core moiety flipping from the inner leaflet to outer leaflet of the inner membrane [24,25], and then Imp/RlpB protein complex was responsible for transport of LPS from the periplasm to the outer leaflet of the outer membrane [17]. Here we showed that *imp/ostA *and *msbA *might be synergistic in hydrophobic drugs resistance and LPS transport in *H. pylori*.

## Methods

### Chemicals

Glutaraldehyde was purchased from Electron Microscopy Sciences (Hatfield, PA). Chloramphenicol, erythromycin, kanamycin, novobiocin, rifampicin, ethidium bromide, and carbonyl cyanide *m*-chlorophenylhydrazone (CCCP) were purchased from Sigma Chemical Co (St Louis, MO).

### Bacterial strains and culture conditions

Clinical isolates were collected from National Taiwan University Hospital (NTUH) as previously described [26]. *H. pylori *strains were grown on Columbia agar plates containing 5% sheep blood under microaerophilic conditions (5% O_2_, 10% CO_2_, and 85% N_2_) at 37°C. For microarray analysis, we selected a rapidly growing strain NTUH-S1 with a higher MIC (MIC = 6 μg/ml) to glutaraldehyde from a patient with gastritis to study gene expression. To screen for mutant strains, blood agar plates were supplemented with 4 μg/ml chloramphenicol or 10 μg/ml kanamycin. To screen for *imp/ostA *and *msbA *double deletion mutant or complementation strains, blood agar plates were supplemented with 4 μg/ml chloramphenicol and 10 μg/ml kanamycin.

### Determination the MICs of glutaraldehyde and hydrophobic drugs in *H. pylori*

The MICs of glutaraldehyde and hydrophobic drugs (erythromycin, novobiocin, rifampicin, and ethidium bromide) were determined by the agar dilution method. Suspension of *H. pylori *was adjusted to 10^7 ^cells/ml. Five microliters of bacterial suspensions were spotted on blood agar plates supplemented with different concentrations of drugs. Results were observed after 72 h incubation under microaerophilic condition at 37°C.

### RNA slot blot hybridization

Four strains with the MICs of 7–10 μg/ml glutaraldehyde (designed numbers 1~4), four with the MICs of 4–6 μg/ml glutaraldehyde (numbers 5~8), and three with the MICs of 1–3 μg/ml glutaraldehyde (numbers 9~11) were grown on Columbia blood agar plates for 48 h, and further passaged on Columbia blood agar plates or 0.5 μg/ml glutaraldehyde-containing blood agar plates for 48 h. Since 0.5 μg/ml was the half concentration of the minimum MIC for the 11 strains, we defined this as the induction concentration. Subsequently, RNA was extracted from the bacteria with or without glutaraldehyde treatment. Total RNA from each *H. pylori *clinical isolate was extracted as described previously [27]. Ten micrograms of total RNA was transferred onto a nylon membrane using a slot-blot system (Hoefer, Holliston, MA). The membrane was hybridized with DNA probes specific for 23S rRNA (0.9 kb PCR-amplified 23S rRNA-specific fragment using the forward primer: 5'-ATTGGAGGGAAGGCAAATCC-3' and the reverse primer: 5'-ATCACTATGACCGACTTTCG-3'), *imp/ostA *(0.8 kb PCR-amplified *imp/ostA*-specific fragment using the forward primer: 5'-CATTGATAACCCCATTTGGC-3' and the reverse primer: 5'-GCACATTCAAAGCGTTTTGC-3'), and *msbA *(0.8 kb PCR-amplified *msbA*-specific fragment using the forward primer: 5'-TAGCGTTAGTGGGGTTAGTC-3' and the reverse primer: 5'-ACACCCTTTGAGTGACAACG-3') labeled with DIG by PCR. Detection was performed with the DIG Luminescent Detection kit (Roche Diagnostics, Indianapolis, IN) according to the manufacturer's instructions.

### RNA isolation and quantitative real-time PCR

It takes 48 to 72 h to recover colonies when *H. pylori *were grown on blood agar plates. A previous report also detected consistent RNA expression levels changes of *H. pylori *after 48 h of growth on acidified blood agar plates [27]. *H. pylori *NTUH-S1 was grown on Columbia blood agar plates for 48 h, and further passaged on Columbia blood agar plates or 0.5 μg/ml glutaraldehyde-containing blood agar plates for 48 h. RNA was extracted by the QIAGEN RNeasy column purification kit (Qiagen, Hilden, Germany) according to the manufacturer's instructions. Total RNA was quantified with a spectrophotometer and visualized on an ethidium bromide stained agarose gel. Total RNA served as a template for cDNA synthesis using the SuperScript II Reverse transcriptase (Invitrogen, Carlsbad, CA). Synthesis reactions were started with 1.5 μg total RNA per 20 μl reaction mixture. All reactions were normalized to the level of the 16S rRNA gene [28]. In real-time RT-PCR, amplification and detection of the cDNAs were monitored using the KAPA SYBR FAST qPCR kit (Kapabiosystems, Boston, MA) in an ABI 7900 thermocycler (Applied Biosystems, Carlsbad, CA). Gene-specific primers *imp/ostA *RT (F): 5'-TTTGTCTTTAGGGCTTTGGAATG-3', *imp/ostA *RT (R): 5'-GCACGAAGGAATTTTTAGATTGC-3' and 16S rRNA RT (F):5'-TGCGAAGTGGAGCCAATCTT-3', 16S rRNA RT (R): 5'-GGAACGTATTCACCGCAACA-3' were used for amplification of cDNAs in this experiment. For the *imp/ostA *gene, the calculated threshold cycle (Ct) was normalized to the Ct of the 16S rRNA gene from the same cDNA sample before the fold change was calculated using the ΔΔCt method as described previously [29].

### Western blots analysis of cell extracts

Eleven strains (numbers 1~11, the same isolates as previously described in RNA slot blot hybridization experiments) were selected and grown on Columbia blood agar plates for 48 h, and further passaged on Columbia blood agar plates or 0.5 μg/ml glutaraldehyde-containing blood agar plates for 48 h. Bacteria were harvested by centrifugation. Cells were washed in phosphate-buffered saline (PBS), resuspended in lysis buffer (50 mM Tris-HCl, 500 mM NaCl, 0.1% SDS, 10% glycerol), and lysed by sonication. Total protein concentration was determined by using the Bio-Rad protein assay (Bio-Rad, Hercules, CA). Samples were loaded at identical protein concentrations from total cell extracts for Western blot analysis. SDS-PAGE was transferred to nitrocellulose for immunological detection. Membrane was blocked with 5% skimmed milk in TBS overnight at 4°C. Subsequently, membrane was incubated with anti-OstA polyclonal antibody [14] diluted 1:500 with 5% skimmed milk in TTBS (0.5% Tween-20) for 1 h at room temperature. Horseradish peroxidase-conjugated anti-rat IgG diluted 1:3000 with 5% skimmed milk in TTBS (0.5% Tween-20) was added and membrane was incubated for 1 h at room temperature. The membrane was washed three times with TTBS (0.5% Tween-20) between the incubation steps. Electrochemiluminescence (Amersham Biosciences, Fairfield, CT) was used for detection.

### RNA isolation and microarray analysis of *H. pylori *NTUH-S1

*H. pylori *NTUH-S1 was grown on Columbia blood agar plates for 48 h and further passaged on Columbia blood agar plates or 3 μg/ml glutaraldehyde-containing blood agar plates for 48 h. RNA was extracted using the QIAGEN RNeasy column purification kit (Qiagen) according to the manufacturer's instructions.

cDNA was synthesized according to the SuperScript™ indirect cDNA Labeling System (Invitrogen). cDNA was then purified using the S.N.A.P column purification (Invitrogen) according to the manufacturer's instructions.

Aminoallyl dUTP-labeled cDNA was resuspended in 2 × coupling buffer and labeled with either Alexa Fluor 555 or 647 according to the manufacturer's protocol (Molecular Probes, Eugene, OR). Labeled cDNA was mixed together and purified by S.N.A.P column purification. Then, the labeled cDNA was concentrated with a Microcon YM-30 column (Millipore, Billerica, MA).

The Institute for Genomic Research (TIGR) provided a *H. pylori *whole-genome microarray. It consisted of 2,572 70-mer oligonucleotides, printed in quadruplicate and representing open reading frames from *H. pylori *26695 and strain J99. Labeled cDNA was resuspended in filtered hybridization buffer (50% formamide, 5 × SSC, 0.1% sodium dodecyl sulfate, 0.1 M DTT, and 0.6 μg/ml salmon sperm DNA), denatured at 95°C for 5 min, and flicked for an additional minute. It was then denatured for another 5 min. The labeled probe was applied to the pre-hybridized microarray and placed in a hybridization chamber at 42°C for 16~20 h. Microarray scanning and analysis were performed on a scanner (GenePix 4000B with GenePix Pro 5.0 software; Axon, Foster City, CA).

Processed microarray data files have been deposited in the Center for Information Biology Gene Expression Database (CIBEX; http://cibex.nig.ac.jp) under accession number CBX86.

### Construction of *imp/ostA *and *msbA *deletion mutants

The gene encoding Imp/OstA with the upstream and downstream 500 bp flanking region was amplified with the genomic DNA of wild-type NTUH-S1 by PCR. The forward primer was 5'-ATGCACTCTCCAAATTTAGA-3', and the reverse primer was 5'-GGGGCTAGGATAGGTTCTAA-3'. It was then cloned into a pGEM-T easy vector (Promega, Madison, WI).

The gene encoding MsbA with the upstream 458-bp and downstream 474-bp flanking regions was amplified with the genomic DNA of wild-type NTUH-S1. PCR was performed using the forward primer, 5'-ACGACAGGAAACCCTTTAGG-3' and the reverse primer was 5'-AGCGTAATAAACAGGCACGC-3'. It was also cloned into a pGEM-T easy vector (Promega).

The *imp/ostA *and *msbA *genes were deleted by inverse PCR, and a chloramphenicol-resistant cassette (a gift from Dr. D. E. Taylor, University of Alberta) with its coding region (from the 1-bp start codon to the 624-bp stop codon) was then cloned into the flanking regions to replace the full-length *imp/ostA *or *msbA *gene. This plasmid was natural transformed into the wild-type NTUH-S1 strain to generate deletion mutants. Chromosomal DNA of the transformants was checked by PCR with primers external and internal to the replacement site to verify the double-crossover event.

### Complementation of *imp/ostA *and *msbA*

An *imp/ostA *complementation strain of NTUH-S1 was constructed as described previously [14]. The promoter site of *msbA *gene was predicted by using a tool available at the following website: http://www.fruitfly.org/seq_tools/promoter.html. The *msbA *gene containing the predicted promoter region (upstream 73 bp) was obtained by PCR using the forward primer: 5'-CCAATCGCTTTAAGCTG-3', and the reverse primer: 5'-TTAGCATTCTGTCAAACGCC-3'. Then the DNA fragment was cloned into the pGEM-T easy vector (Promega). The *msbA *gene with its promoter region was cut from the constructed pGEM-T easy vector and ligated into the NruI site of the shuttle vector pHel3 (plasmid pHel3 was a gift from Dr. R. Haas, Max-Planck-Institute für Biologie, Tübingen, Germany). The constructed shuttle vector was natural transformed into an *msbA *deletion mutant strain to generate the *msbA *complementation strain.

### Construction of the *imp/ostA *and *msbA *double deletion mutant

The gene encoding MsbA with its upstream 458-bp and downstream 474-bp flanking region was cloned into the pGEM-T easy vector as described above. A kanamycin-resistant gene *aphA-3 *from *Campylobacter jejuni *was then cloned between the flanking regions to replace the full length *msbA *gene. This plasmid was natural transformed into the wild-type NTUH-S1 strain to generate the deletion mutant. Chromosomal DNA of the transformants was checked by PCR with primers external and internal to the replacement site to verify the double-crossover event. Then, chromosomal DNA from *msbA *deletion mutant strain (Km^r^) was natural transformed into the *imp/ostA *deletion mutant to obtain a double deletion mutant strain. It was also confirmed by PCR with primers external and internal to the *msbA *gene replacement site.

### Southern blotting

Approximately 5 μg of genomic DNA from *H. pylori *NTUH-S1 and the mutants was digested by Hind III and incubated at 37°C overnight for complete digestion. The digoxigenin-labeled *imp/ostA *and *msbA *probes (primers were the same as those described for slot blot) was generated by PCR. Hybridization and detection were performed with the DIG Luminescent Detection kit (Roche) according to the manufacturer's instructions.

### Proteinase-K digested *H. pylori*

The procedure was followed as described previously [30]. *H. pylori *cells were collected and adjusted to a concentration of 2.5 × 10^9 ^cells/ml in PBS. Bacteria were boiled with 150 μl sample dye for 10 min at 100°C to disrupt the whole cells. Subsequently, the whole cell lysates were treated with proteinase K (Sigma) for 60 min at 60°C in a water bath. Then, 2.5 × 10^8 ^cells/ml were analyzed by 12% SDS-PAGE and stained with silver. The protein concentration of the 2.5 × 10^8 ^cells/ml was also determined by using the Bio-Rad protein assay (Bio-Rad) to serve as a loading control.

### Immunoblots of LPS from *H. pylori *with anti-Lewis (Le) monoclonal antibody

*H. pylori *strains that have been screened serologically [31-33], and a previous study suggested that Asian isolates express predominantly type 1 (Le^a^, Le^b^) antigens compared to Western strains (predominantly expressing type 2 Le^x ^and Le^y ^determinants) [34]. We also primarily detected the Lewis antigen of NTUH-S1 with anti-Le^a ^and anti-Le^b ^antibody. Equivalent amounts of protein were loaded in each well and transferred to nitrocellulose for immunological detection with anti-Le^a ^or anti-Le^b ^monoclonal antibody (Seikagaku Corporation, Tokyo). For detection of Lewis antigen in proteinase K-digested whole cell lysates, nitrocellulose membrane was blocked with 5% skimmed milk in PBS for 1 h at room temperature. Subsequently, membrane was incubated with anti-Le^a ^or anti-Le^b ^antibody diluted 1:3000 with 5% skimmed milk in PBS overnight at 4°C. Horseradish peroxidase-conjugated anti-mouse IgG diluted 1:5000 with 5% skimmed milk in PBS was added and membrane was incubated for 1 h at room temperature. The membrane was washed three times with PBST (0.05% Tween-20) between the incubation steps. Electrochemiluminescence (Amersham Biosciences) was used for detection. Whole cells of the Le^x ^and Le^y ^antigen-expressing *H. pylori *26695 strain [35] were used as a negative control in Western blots to ensure the specificity of the anti-Le^a ^or anti-Le^b ^antibody.

### Measurement of outer membrane permeability by ethidium bromide

Outer membrane permeability can be measured by the fluorescence of the ethidium-polynucleotide complex in the cell because ethidium bromide displays approximately a 10-fold increase in fluorescence quantum yield upon binding to DNA [36]. The assay was modified as described previously [37]. Briefly,*H. pylori *were grown on Columbia blood agar plate for 48 h. Then, bacteria were pelleted and washed twice with ice-cold 50 mM potassium phosphate (pH 7.0) containing 5 mM MgSO_4_. Cells were resuspended in 1 ml of potassium phosphate buffer (pH 7.0) at an optical density (OD_600_) of 0.5 and incubated for 30 min at 37°C in the presence of 10 μM of CCCP to deplete cells of metabolic energy. Subsequently, cells were washed three times in ice-cold potassium phosphate (pH 7.0) containing 5 mM MgSO_4 _and loaded with 10 μg/ml ethidium bromide. At the 40-min time point, the increase in ethidium bromide fluorescence intensity was measured in Gemini XPS spectrofluorimeter (Molecular Devices, Sunnyvale, CA) at 30°C with the excitation wavelength set at 500 nm and the emission wavelength at 580 nm. Each measurement was repeated three times.

### Ethidium bromide accumulation assay

The assay was modified as described previously [38]. Briefly,*H. pylori *were grown on Columbia blood agar plate for 48 h. Then, bacteria were pelleted and washed twice with ice-cold 50 mM potassium phosphate (pH 7.0) containing 5 mM MgSO_4_. Cells were resuspended in 1 ml of potassium phosphate buffer (pH 7.0) at an optical density (OD_600_) of 0.5. Cells were preloaded with 10 μg/ml ethidium bromide. At the 12-min time point, 10 μM of CCCP was added to the cells suspensions to assess energy-dependent efflux. CCCP was not added to the cells served as a control. The increase in ethidium bromide fluorescence intensity was measured in a Gemini XPS spectrofluorimeter at 30°C with excitation at 500 nm and emission at 580 nm. Each measurement was repeated three times.

### Statistical analysis

For all experiments, a *P *value of < 0.05 was considered indicative of statistical significance, and all statistical analyses were determined with Student's *t *test.

## Results

### The MICs for glutaraldehyde in clinical isolates

**Figure 1:**
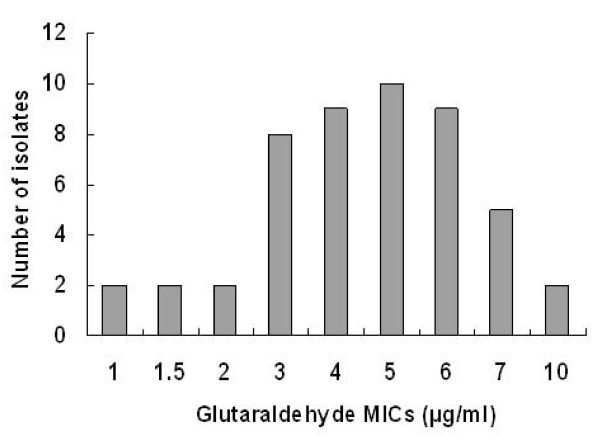
**The MICs of glutaraldehyde in clinical isolates from National Taiwan University Hospital**.

*H. pylori *strains were harvested during endoscopic examinations at National Taiwan University Hospital from 1991 to 2000 [39]. 49 clinical isolates were cultured successfully from stock and stored at -80°C. The patients from which these strains were isolated suffered from gastritis (15 strains), duodenal ulcer (16 strains), gastric ulcer (9 strains), mucosa-associated lymphoid tissue lymphoma (MALToma) (3 strains), and gastric cancer (6 strains). Subsequently, the MICs of glutaraldehyde were determined for these strains. The MICs of glutaraldehyde for most of the clinical isolates were the range of 3–6 μg/ml glutaraldehyde (Fig. 1). However, the diseases caused by the strains of *H. pylori *and the MICs of glutaraldehyde in these clinical isolates were not correlated (Table 1).

**Table 1:** The MICs of glutaraldehyde in clinical isolates during 1991–2000.

Disease	Number of isolates	The MICs of glutaraldehyde in isolates (numbers)
Gastritis	15	7 μg/ml (n = 2), 6 μg/ml (n = 1) 5 μg/ml (n = 3), 4 μg/ml (n = 4) 3 μg/ml (n = 5)

Duodenal ulcer	16	10 μg/ml (n = 1), 7 μg/ml (n = 1) 6 μg/ml (n = 2), 5 μg/ml (n = 3) 4 μg/ml (n = 5), 2 μg/ml (n = 2) 1.5 μg/ml (n = 1), 1 μg/ml (n = 1)

Gastric ulcer	9	10 μg/ml (n = 1), 7 μg/ml (n = 1) 6 μg/ml (n = 3), 5 μg/ml (n = 1) 3 μg/ml (n = 1), 1.5 μg/ml (n = 1) 1 μg/ml (n = 1)

MALToma	3	7 μg/ml (n = 1), 5 μg/ml (n = 2)

Gastric cancer	6	6 μg/ml (n = 3), 5 μg/ml (n = 1) 3 μg/ml (n = 2)

### The RNA and protein expression levels of *imp/ostA *in clinical isolates after glutaraldehyde treatment

**Figure 2:**
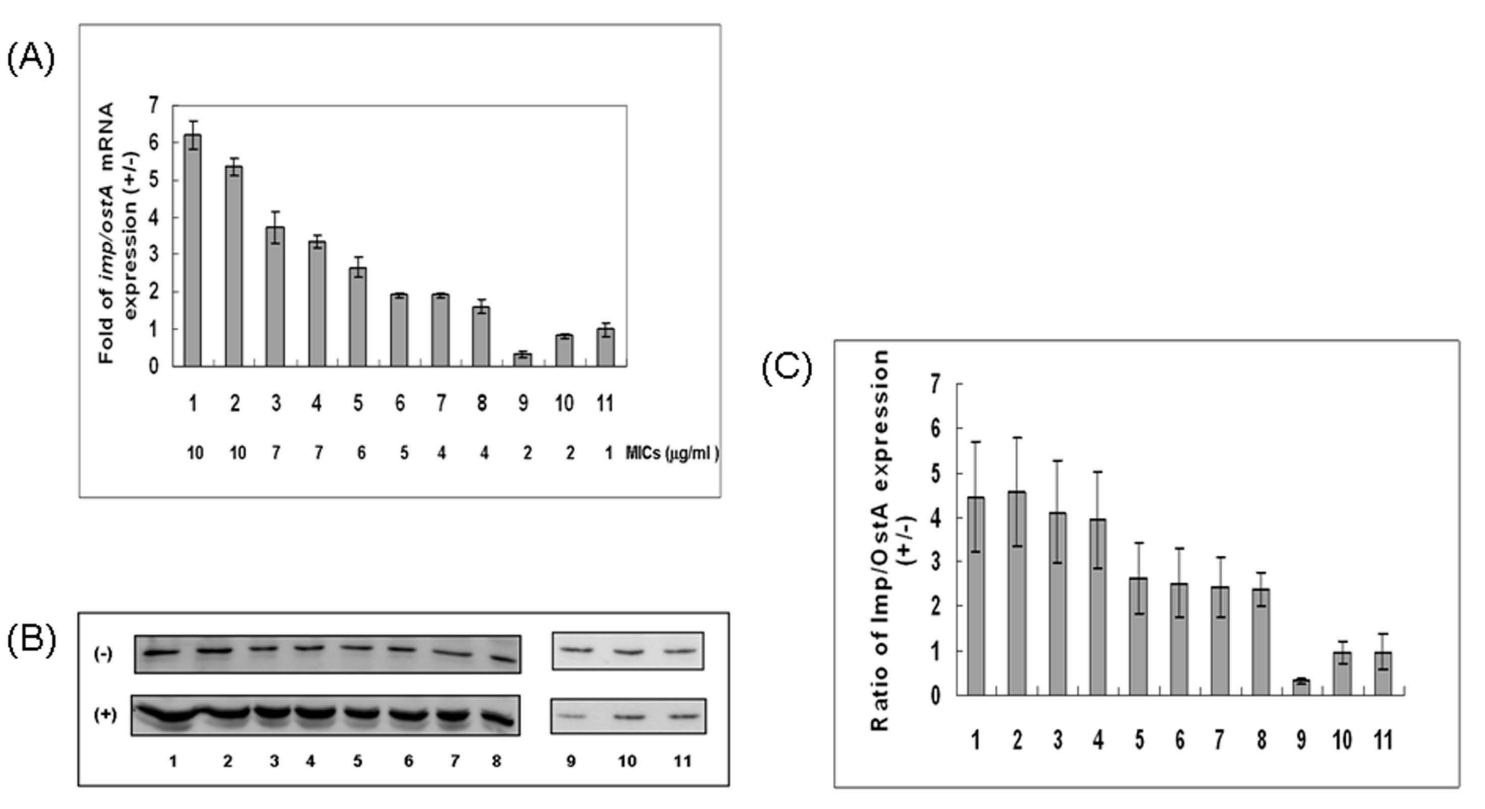
**The RNA and protein expression levels of *imp/ostA *in clinical isolates after glutaraldehyde treatment**. (A) Quantitative real-time PCR analysis of the relative expression of *imp/ostA *mRNA after glutaraldehyde treatment in 11 clinical isolates. The MICs of the corresponding strains are shown in the lower portion of the figure. Each bar represents the relative expression after glutaraldehyde treatment. (B) Western blot analysis of Imp/OstA protein expression. (+) represents glutaraldehyde treatment; (-) represents no glutaraldehyde treatment. (C) The ratio of Imp/OstA protein expression with and without glutaraldehyde treatment. The results were from three independent experiments.

In our previous study [14], we found that the barrier function of the outer membrane against drugs might be decreased in the *imp/ostA *mutant strain, suggesting that glutaraldehyde may enter the mutant strain more rapidly than it enters the wild-type strain. Four strains with the MICs of 7–10 μg/ml (designed numbers 1~4), four with the MICs of 4–6 μg/ml (numbers 5~8), and three with the MICs of 1–3 μg/ml (numbers 9~11) were selected to clarify the correlation of *imp/ostA *expression with glutaraldehyde resistance. Subsequently, RNA was extracted from bacteria after 48 h with or without 0.5 μg/ml glutaraldehyde treatment. However, RNA expression of *imp/ostA *in strains without glutaraldehyde treatment was not detected by slot blot (data not shown). Therefore, we further examined RNA expression of *imp/ostA *by quantitative real-time PCR. The result indicated that RNA expression of *imp/ostA *induced by glutaraldehyde was higher in strains with the MICs of 4–10 μg/ml than that in strains with the MICs of 1–3 μg/ml (*P*= 0.001455) (Fig. 2A). Expression of Imp/OstA protein in these 11 strains after glutaraldehyde treatment was also examined (Fig. 2B). The intensity of protein expression in three independent experiments was analyzed by Image Quant 5.1, and the ratio of Imp/OstA protein expression in the 11 strains with and without glutaraldehyde treatment was calculated. The ratio of Imp/OstA expression induced by glutaraldehyde was higher for strains with the MICs of 4–10 μg/ml (numbers 1~8) than strains with the MICs of 1–3 μg/ml (numbers 9~11) (*P *= 6.1 × 10^-5^) (Fig. 2C). These results suggested that the expression of *imp/ostA *and Imp/OstA was involved in glutaraldehyde resistance in clinical isolates after glutaraldehyde treatment.

### Full genome expression after glutaraldehyde treatment

We next examined the alterations in RNA expression in *H. pylori *NTUH-S1 induced by glutaraldehyde. After treatment with glutaraldehyde for 48 h, 40 genes were upregulated at least 2.5-fold, and 31 genes were downregulated at least 2.5-fold (see Additional File 1), compared to the untreated bacteria. The upregulated genes included *imp/ostA*, which was upregulated 9.218-fold. These results are in agreement with the quantitative real-time PCR data, showing that this gene was notably expressed after glutaraldehyde treatment. Another interesting finding was that glutaraldehyde upregulated another lipid transport gene *msbA *(HP1082) by 2.661-fold. Previous reports indicated that this subfamily of ABC transporters is involved in transport of many different substrates, such as peptides, lipids, hydrophobic drugs, polysaccharides, and proteins [40]. MsbA is a lipid flippase that transports the lipid A-core moiety from the inner to the outer leaflet of the inner membrane in *E. coli *[17,41]. Imp/OstA also participates in transport of LPS to the cell surface in *E. coli *[17] and *N. meningitidis *[20]. We proposed that MsbA might be correlated with LPS transport in *H. pylori*. The deficiency in a LPS biosynthesis gene could result in antibiotic susceptibility, especially for hydrophobic antibiotics [42-44]. Therefore, weregarded *msbA *as a suitable candidate for investigating glutaraldehyde or other hydrophobic drug transport in bacteria.

### Reconfirmation of *msbA *expression in the clinical isolates by slot blots hybridization

**Figure 3:**
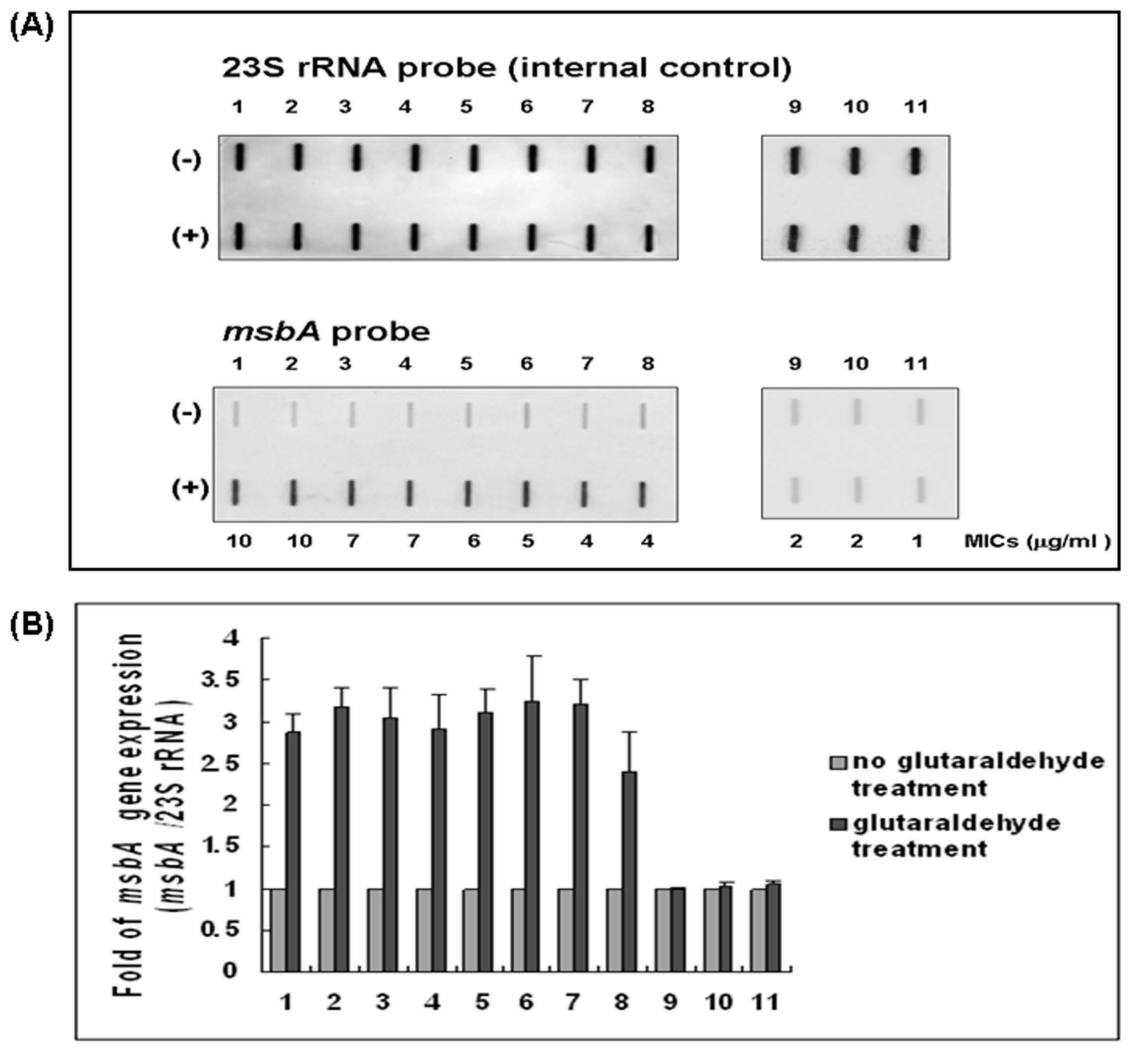
**The expression of *msbA *in 11 clinical isolates**. (A) Slot blots analysis of *msbA *expression in 11 clinical isolates. Hybridization was performed with DIG probes specific for 23S rRNA and *msbA*. (+) represents glutaraldehyde treatment. (-) represents no glutaraldehyde treatment. (B) Bacteria were treated or not treated with glutaraldehyde by three independent experiments. The RNA ratio (*msbA*/23S rRNA) without glutaraldehyde treatment was defined as 1, and the RNA ratio with glutaraldehyde treatment was calculated.

Microarray analysis demonstrated that *msbA *was upregulated by glutaraldehyde treatment, and the level of *msbA *expression in the clinical isolates after glutaraldehyde treatment was further determined by slot blot. RNA from the 11 strains used in the *imp/ostA *expression experiment (numbers 1~11) was extracted before or after glutaraldehyde treatment and hybridized with probes specific for 23S rRNA or *msbA*. The *msbA *transcripts were weakly detectable in the control without glutaraldehyde treatment; therefore, the RNA ratio (*msbA*/23S rRNA) without glutaraldehyde treatment was defined as 1, and the RNA ratio with glutaraldehyde treatment was calculated. The results confirmed the increased expression of *msbA *induced by glutaraldehyde (Fig. 3A). Furthermore, the level of *msbA *expression induced by glutaraldehyde was higher in strains with the MICs of 4–10 μg/ml than that in strains with the MICs of 1–3 μg/ml (*P *= 6.63 × 10^-8^) (Fig. 3B).

### Effect of *imp/ostA *on the transcription of msbA after glutaraldehyde treatment

**Figure 4:**
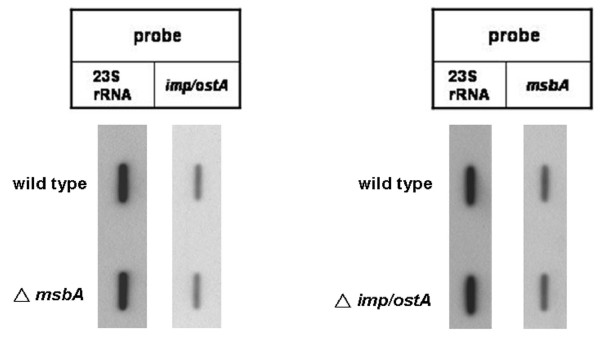
**The effect of *imp/ostA *on the transcription of *msbA *after glutaraldehyde treatment and vice versa**. Slot blots analysis of total RNA preparations of *H. pylori *NTUH-S1 wild-type and mutants after 0.5 μg/ml glutaraldehyde treatment for 48 h. Each well was loaded with 10 μg total bacterial RNA. The membrane was hybridized with DIG-labeled probes specific for *H. pylori imp/ostA*, *msbA*, and 23S rRNA.

The expression of both *imp/ostA *and *msbA *was increased in NTUH-S1 after glutaraldehyde treatment according to the results of the microarray analysis. To determine whether *imp/ostA *affects *msbA *gene expression after glutaraldehyde treatment and vice versa, RNA levels of *imp/ostA *and *msbA *in wild-type and mutant strains after 0.5 μg/ml glutaraldehyde treatment were analyzed by slot blot. The *imp/ostA *transcript was not affected in the *msbA *deletion mutant in comparison with the wild-type strain after glutaraldehyde treatment (Fig. 4). Likewise, the *msbA *transcript was not affected in the *imp/ostA *deletion mutant in comparison with the wild-type strain after glutaraldehyde treatment. This result indicated that *imp/ostA *and *msbA *were induced by glutaraldehyde through independent pathways.

### The MICs of glutaraldehyde in isogenic mutants

**Figure 5:**
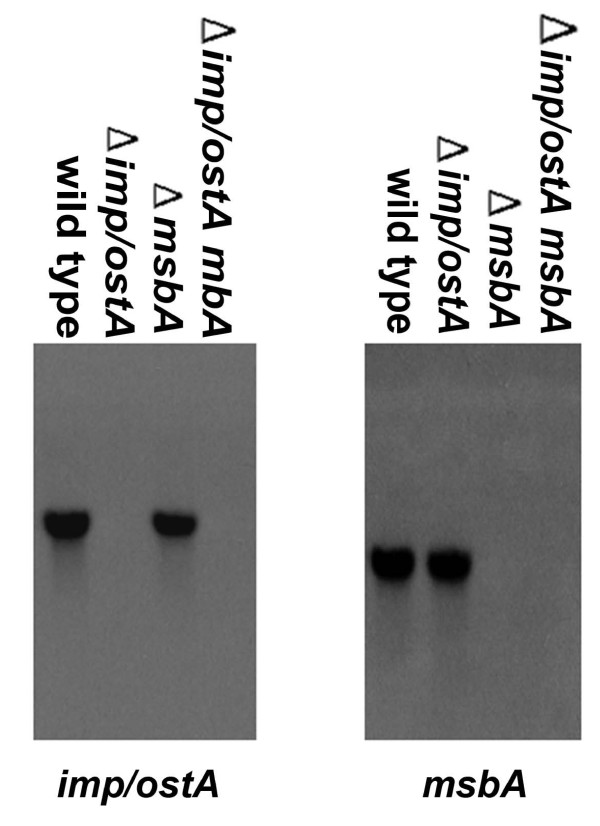
**Southern hybridization of Hind III-digested DNA from strains NTUH-S1 and mutants with *imp/ostA *(left) and *msbA *(right) probes**. Approximately 5 μg of genomic DNA from *H. pylori *NTUH-S1 and the mutants was digested by Hind III. Hybridization and detection were performed with the DIG Luminescent Detection kit (Roche) according to the manufacturer's instructions.

**Figure 6:**
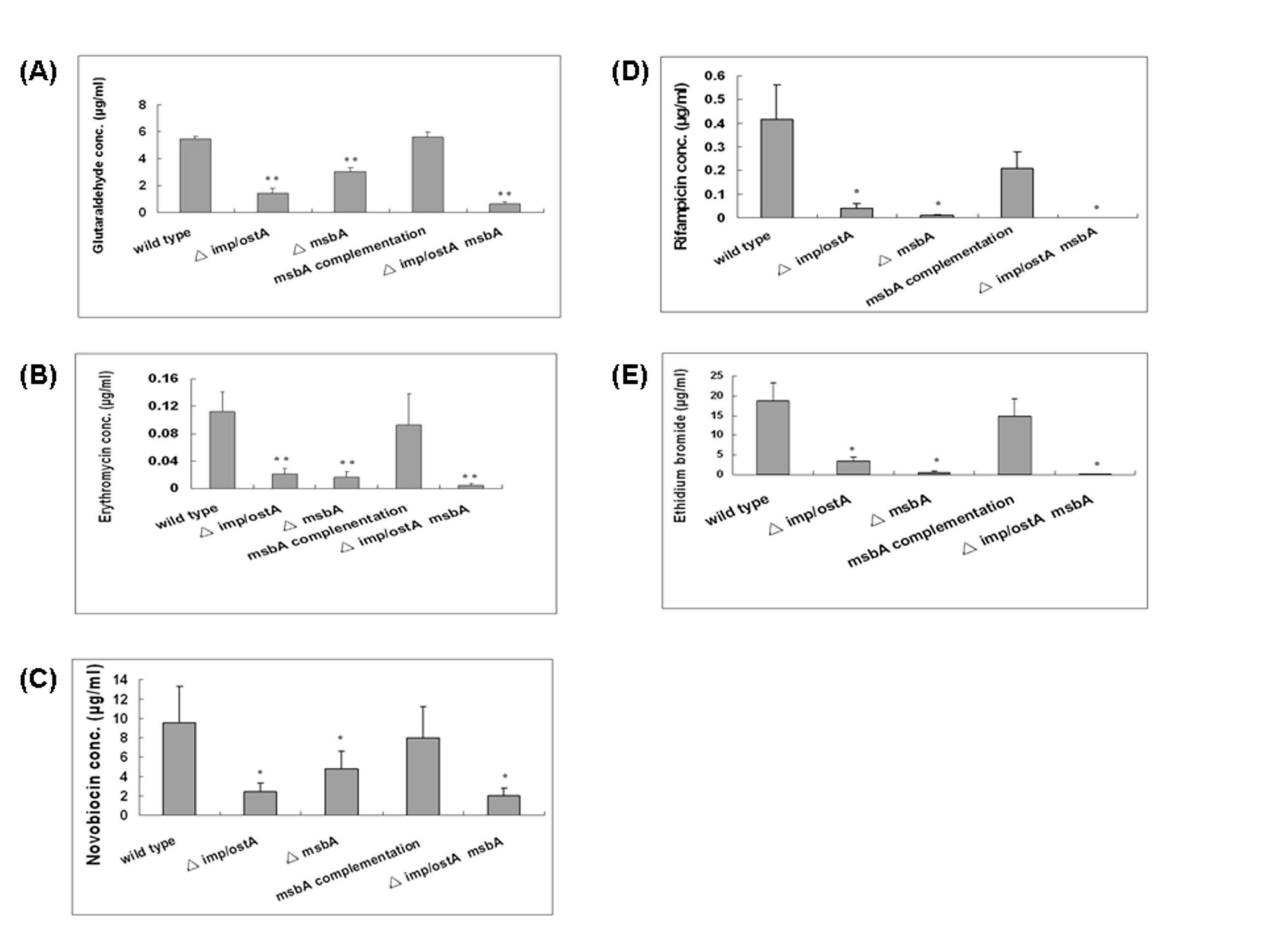
**Determination of the MICs glutaraldehyde, erythromycin, novobiocin, rifampicin, and ethidium bromide in *H. pylori *and isogenic mutants (A-E)**. Experiments were repeated three to five times. *, *P *< 0.05 vs. wild-type, and **, *P *< 0.001 vs. wild-type. Error bars indicate the standard deviation.

We had previously observed that the *imp/ostA *mutant became more sensitive to glutaraldehyde than wild-type strain [14]. Southern blot hybridizations were performed to confirm that *imp/ostA *or *msbA *were absent in the mutants (Fig. 5). We further investigated whether the sensitivities to glutaraldehyde ofisogenic *msbA *and an *imp/ostA*, *msbA *double mutants were altered. The MIC for the *msbA *single mutant (3.05 ± 0.27 μg/ml) was lower than for wild-type (5.45 ± 0.21 μg/ml) (wild-type vs.*msbA *single mutant, *P *= 2.84 × 10^-7^). For comparison, the MIC for the *imp/ostA *single mutant (1.40 ± 0.42 μg/ml) was also significantly lower than that of wild-type, as previously reported [14]. Furthermore, the MICs for *imp/ostA *and *msbA *double mutant (0.60 ± 0.14 μg/ml) was also significantly lower than that of wild-type and showed the most significant difference (*P *= 5.77 × 10^-10^). Complementation of the *msbA *mutation significantly restored the resistance to glutaraldehyde (Fig. 6A). These results suggested that *imp/ostA *and *msbA *were both involved in glutaraldehyde resistance, and the deficiency of these two genes in *H. pylori *led to hypersensitivity to glutaraldehyde.

### The MICs of hydrophobic antibiotics in isogenic mutants

According to previous reports [41,45], MsbA interacts with multiple drugs, for example, multidrug resistance (MDR) substrates (doxorubicin, vinblastine, erythromycin, ethidium bromide) and non-MDR substrates (lipid A, Hoechst). In addition, MsbA increases resistance to erythromycin by 86-fold when it is expressed in *L. lactis *[22]. In contrast, expression of MsbA in *Pseudomonas aeruginosa *did not confer resistance to erythromycin, but introducing *E. coli msbA *into *P. aeruginosa *decreased the susceptibility of this bacterium to erythromycin by 4-fold [46]. Hence, we investigated whether the isogenic *msbA *mutant was more sensitive to erythromycin than wild-type *H. pylori*. The result showed that the MICs were 0.112 ± 0.029 μg/ml and 0.017 ± 0.008 μg/ml for wild-type and the *msbA *deletion mutant (wild-type vs. *msbA *deletion mutant, *P*= 0.00059, respectively). This indicated that MsbA participated in erythromycin resistance in *H. pylori*. In a previous study [14], it has been reported that the mutation of *imp/ostA *resulted in a lower MIC of erythromycin in *H. pylori*. In this study, deletion of both *imp/ostA *and *msbA *enhanced the susceptibility to erythromycin (*P*= 0.00055) (Fig. 6B).

Previous reports demonstrated that in Gram-negative bacteria, a deficiency of the LPS biosynthesis gene would result in antibiotic susceptibility, especially for hydrophobic antibiotics [42-44]. Therefore, we determined the MICs of two hydrophobic antibiotics, novobiocin and rifampicin, to investigate whether *imp/ostA *and *msbA *participated in resistance to hydrophobic antibiotics. Both *imp/ostA *and *msbA *single mutants were more sensitive to novobiocin and rifampicin than wild-type (Fig. 6C and 6D). These results indicated that imp/ostA and msbA are important for H. pylori resistence to hydrophobic antibiotics. The MIC of rifampicin for the *imp/ostA *and *msbA *double mutant (0.00037 ± 0.00013 μg/ml) decreased significantly.

In order to determine the transport route of hydrophobic drugs in bacteria, the hydrophobic compound ethidium bromide was used. In this way, the MIC of ethidium bromide for *H. pylori *was also examined. The result showed that the *msbA *mutant was more susceptible to ethidium bromide than the wild-type strain. This result suggests that MsbA might be involved in active efflux by *H. pylori*, as evidenced by an approximately 36-fold reduction in the MIC of the *msbA *mutant compared to the wild-type strain (Fig. 6E).

### LPS production in *H. pylori *wild-type and isogenic mutants

**Figure 7:**
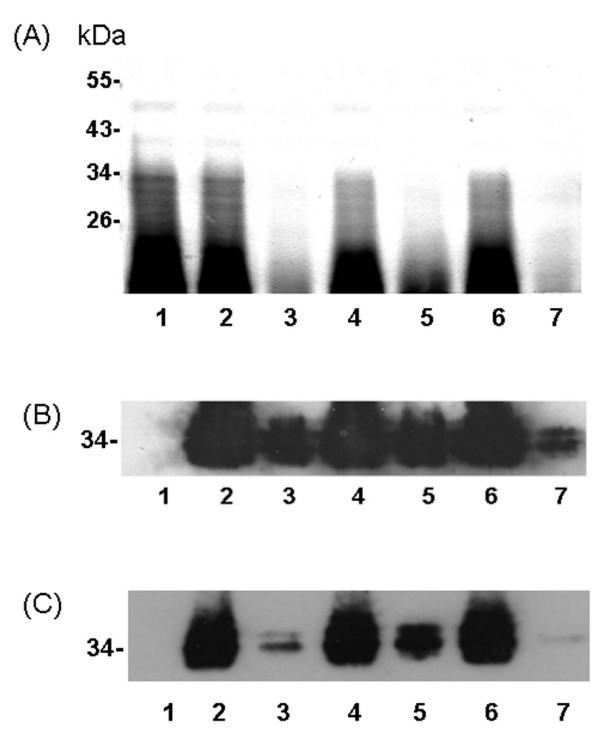
**Silver-stained of proteinase-K digested whole cell lysate from *H. pylori *wild-type and isogenic mutants**. (A) Lanes 1–7 were all loaded with 2.5 × 10^8 ^proteinase K-digested bacteria (~130 μg total protein). Lane 1, 26695; lane 2, wild-type; lane 3, *imp/ostA *single mutant strain; lane 4, *imp/ostA *complementation strain; lane 5, *msbA *single mutant strain; lane 6, *msbA *complementation strain; lane 7,*imp/ostA *and *msbA *double mutant strain. Molecular weights of the prestained markers are indicated. (B-C) Immunoblots of LPS from *H. pylori *with anti-Le^a ^or anti-Le^b ^monoclonal antibodies. (B) anti-Le^a ^(1:3000) as the primary antibody and anti-mouse IgG (1:5000) as the secondary antibody, or (C) anti-Le^b ^(1:3000) as the primary antibody and anti-mouse IgG (1:5000) as the secondary antibody.

To investigate whether *imp/ostA *and *msbA *participated in LPS biogenesis, the equivalent amounts of proteinase K-digested whole cells were analyzed by silver staining (Fig. 7A). The total amount of LPS was drastically reduced in the *imp/ostA *single mutant compared with that in the wild-type strain (Fig. 7A, lane 3). This result indicated that *imp/ostA *participated in LPS biogenesis and is consistent with a similar finding in *N. meningitidis *[20]. Mutation of *msbA *decreased LPS production, although small amounts of LPS could be detected (Fig. 7A, lane 5). Furthermore, deletion of both *imp/ostA *and *msbA *severely reduced LPS production. The LPS in *H. pylori *was detected by using anti-Le^a ^(Fig. 7B) or anti-Le^b ^antibody (Fig. 7C). *H. pylori *26695 strain expressing Le^x ^and Le^y ^but not Le^a ^or Le^b ^antigens was used as a negative control to ensure the specificity of the anti-Le^a ^or anti-Le^b ^antibody (Fig. 7B and 7C, lane 1). The data indicated that the NTUH-S1 strain expressed both the Le^a ^and Le^b ^antigens. In addition, the amounts of O-antigen (~34 kDa) in the *imp/ostA *or *msbA *single mutant were reduced, and it was especially reduced in the *imp/ostA *and *msbA *double mutant. The growth curves of the wild-type and mutant strains were also examined, and the growth rates of these mutants did not differ from that of the wild-type strain (data not shown). This result demonstrated that both *imp/ostA *and *msbA *were involved in the production of LPS.

### Outer membrane permeability to ethidium bromide

**Figure 8:**
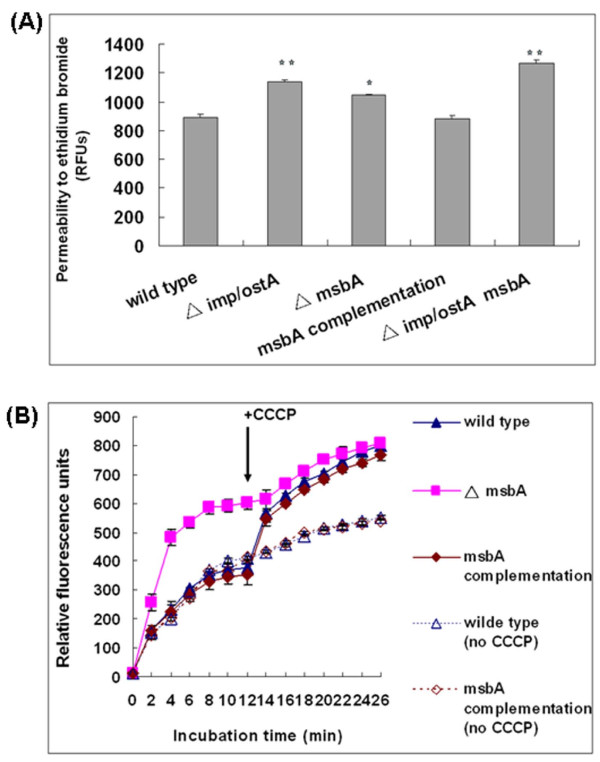
**Permeability and efflux of ethidium bromide**. (A) Determination of the outer membrane permeability in *H. pylori *wild-type and isogenic mutants. Each measurement was repeated three times. *, *P *< 0.05 vs. wild-type, and **, *P *< 0.001 vs. wild-type. (B) Ethidium bromide accumulation assay. Cells were preloaded with 10 μg/ml ethidium bromide. At the 12-min time point, 10 μM of CCCP was added to the cells suspensions to assess energy-dependent efflux. CCCP was not added to the cells serving as controls (dotted lines). Each measurement was repeated three times.

To investigate whether the permeability of the outer membrane was altered in the mutant strains, we measured the fluorescence intensity at a 40-min time point after addition of ethidium bromide and CCCP (Fig. 8A). The fluorescence intensity of the *imp/ostA *deletion mutant (1142.73 ± 12.38 relative fluorescence units [RFUs]) was higher than that of the wild-type (891.29 ± 20.62 RFUs, *P *= 0.0001). The fluorescence intensity of the *msbA *deletion mutant was also significantly higher than the wild-type (*P *= 0.00164). These results might due to the increase of outer membrane permeability when *imp/ostA *or *msbA *was mutated. Furthermore, the fluorescence intensity of the *imp/ostA *and *msbA *double mutant was also significantly higher than that of wild-type (*P *= 5.83 × 10^-5^). Therefore, the increased sensitivity to hydrophobic compounds conferred by *imp/ostA *and *msbA *mutations can be explained by the enhanced membrane permeability for the toxic substances moving in.

### The role of *msbA *in ethidium bromide efflux

As ethidium bromide is a hydrophobic aromatic compound, we used this compound to mimic glutaraldehyde or hydrophobic antibiotics moving in and efflux. The Ethidium bromide accumulation assay was performed to determine whether the *msbA *deletion mutant was more susceptible to glutaraldehyde or hydrophobic antibiotics due to the loss of an active efflux mechanism. The result showed that the *msbA *deletion mutant accumulated more amounts of ethidium bromide than wild-type (Fig. 8B). When CCCP was added to the cells containing ethidium bromide at 12 min, the accumulation of ethidium bromide increased in wild-type and reached to the level almost equal to that of *msbA *deletion mutant. This indicated that ethidium bromide was retained in the cells when efflux was blocked after the collapse of the cells' metabolic energy by CCCP. In contrast, CCCP had no significant effect on the level of ethidium bromide accumulated in the *msbA *deletion mutant. In addition, ethidium bromide accumulation in the *msbA *complementation strain reached a level almost equal to that of wild-type. CCCP was not added to wild-type or complementation strain containing ethidium bromide at 12 min served as a control. These data indicated that MsbA was involved in hydrophobic drug efflux and that it pumped out ethidium bromide in an energy-dependent process. We concluded that MsbA might pump out glutaraldehyde or hydrophobic antibiotics through an active efflux mechanism in *H. pylori*.

## Discussion

We previously identified that *imp/ostA *was associated with glutaraldehyde resistance in a clinical *H. pylori *strain [14]. In order to further investigate the mechanism of glutaraldehyde resistance, the MICs and the levels of *imp/ostA *expression in clinical isolates were monitored. The result indicated that RNA and protein expression of *imp/ostA *induced by glutaraldehyde was higher in strains with the MICs of 4–10 μg/ml than that in strains with the MICs of 1–3 μg/ml. According to these results, we suggested that *imp/ostA *expression was correlated with glutaraldehyde resistance in *H. pylori *clinical isolates.

After treating NTUH-S1 with glutaraldehyde, 40 genes were found to be upregulated at least 2.5-fold by microarray analysis. For 14 of these genes, DNA or protein sequence alignment yielded no information about their function. The other genes could be divided into three groups: transporters, biosynthesis and metabolism genes, and motility and chemotaxis genes. Two genes were related to iron transport; nonheme iron-containing ferritin (HP0653, *pfr*), which participates in iron metabolism and in gastric colonization by *H. pylori *[47]; and an iron dicitrate ABC transporter (HP0889, *fecD*). Genes including *aimF*, *bioC*, *ispB*, NADH-flavin oxidoreductase (HP0642), and cytochrome c551 peroxidase (HP1461) were involved in biosynthesis and metabolism. Lastly, flagellar hook-associated protein 1 (HP1119, *flgK*) [48] and flagellar hook-associated protein 2 (HP0752, *fliD*) [49,50] were related to motility and chemotaxis. However, these genes might not be directly involved in resistance to glutaraldehyde, and their association with glutaraldehyde resistance needs further investigation. In addition, 31 genes were downregulated at least 2.5-fold after glutaraldehyde treatment. Several adjacent genes seemed to be co-regulated, which is indicative of operon structures. For example, HP0690-HP0693 [51] participated in fatty acid metabolism in the TCA cycle. HP0695-HP0696 [51] participated in hydantoin utilization. In addition, some genes are transcribed at different loci but are involved in outer-membrane composition, which included *hopG*, *hofH*, and *homA*. Lastly, two subunits of the 2-oxoglutarate oxidoreductase, *oorB *and *oorD *[52], are also involved in the TCA cycle for energy metabolism. The correlation between TCA cycle-related genes and glutaraldehyde resistance also needs to be investigated further.

Silver staining revealed that both imp/ostA and msbA participated in the biogenesis of LPS in H. pylori. Similarly mutation of the E. coli LPS biosynthesis gene, lpxA2, resulted in extreme susceptibility to antibiotics, especially hydrophobic antibiotics [42-44]. Therefore, mutation of the LPS biosynthesis genes, *imp/ostA *and *msbA*, might account for the reduction of the MICs for hydrophobic antibiotics.

In the beginning, we observed that the MICs of two glutaraldehyde-resistant strains were 10 μg/ml glutaraldehyde. In fact, this is the half concentration used in our hospital for disinfection during endoscopy. We proposed that some bacteria could survive at the low concentrations in the glutaraldehyde-treated endoscopic environment. According to the MICs tests, LPS analysis, outer membrane permeability assay, and ethidium bromide accumulation assay, the increased sensitivity to hydrophobic compounds conferred by mutations of *imp/ostA *and *msbA *can be explained by the defect in LPS production and increased outer membrane permeability. In addition, the increased sensitivity to hydrophobic compounds conferred by mutation of *msbA *might to the result of accumulation of chemicals that are not pumped out by the MsbA efflux pump. The combination of these effects of the *imp/ostA *and *msbA *would reduce the MICs of cells toward glutaraldehyde and hydrophobic antibiotics. These findings might help us to understand the mechanism of bacterial tolerance to chemical disinfectant and hydrophobic drugs.

## Conclusion

The expression levels of *imp/ostA *and *msbA *were correlated with glutaraldehyde resistance in clinical isolates after glutaraldehyde treatment. Imp/OstA and MsbA play an important role in hydrophobic drugs resistance and LPS biogenesis in *H. pylori*.

## Authors' contributions

HC, TL, and JW conceived and designed the experiments. HC carried out the experiments, analyzed the data, and drafted the manuscript. JY provided clinical isolate strains. TL and JW modified the manuscript. All the authors have read and approved the final manuscript.

## Supplementary Material

Additional file 1 **microarray data**. Genes were upregulated and downregulated after glutaraldehyde treatment by microarray analysis Click here for file
