# Supplementary material for: Synergistic effect of imp/ostA and msbA in hydrophobic drug resistance of Helicobacter pylori
Source: BMC Microbiol. 2009 Jul 13;9:136. doi: 10.1186/1471-2180-9-136 (PMC2719649; doi:10.1186/1471-2180-9-136)
Supplement: Additional file 1 — microarray data. Genes were upregulated and downregulated after glutaraldehyde treatment by microarray analysis [file 1471-2180-9-136-S1.pdf]

Supplementary Table

Table S1. Genes were upregulated after glutaraldehyde treatment*

Cellular role category	Fold change	ORF HP no.†	ORF JHP no.†	Common name and function ♯
Hypothetical	19.818	HP1004	N/A	Hypothetical protein
Transporter	13.700	HP0653	JHP0598	Nonheme iron-containing ferritin, *pfr*
Conserved hypothetical	9.218	HP1216	JHP1138	Conserved hypothetical secreted protein, *imp/ostA*
Amino acid biosynthesis	8.968	HP0652	JHP0597	Phosphoserine phosphatase, *serB*
Hypothetical	7.341	HP1440	JHP1333	Hypothetical protein
Energy metabolism	7.321	HP1238	JHP1159	Formamidase, *aimF*
Transporter	6.848	HP0715	JHP0653	ABC transporter, ATP-binding protein
Hypothetical	6.673	HP0412	JHP0970	Hypothetical protein
Hypothetical	5.662	HP0365	N/A	Hypothetical protein
Biosynthesis and metabolism	5.015	HP0642	JHP0586	NAD(P)H-flavin oxidoreductase, *frxA*
Cell envelope	4.959	HP0651	JHP0596	Alpha1,3-fucosyltransferase, *futB*
Hypothetical	4.937	HP1502	JHP1395	Hypothetical protein
Chaperone	4.488	HP0109	JHP0101	Chaperone and heat shock protein 70, *dnaK*
Transporter	4.429	HP0889	JHP0822	Iron(III) dicitrate ABC transporter, permease protein, *fecD*
Cell envelope	4.414	HP0057	JHP0049	Lipoprotein, putative
Cell envelope	4.397	HP0511	N/A	Lipoprotein Lpp, an urease enhancing factor
Hypothetical	4.282	HP0641	JHP0584	Hypothetical protein
Regulatory	4.232	HP0111	JHP0103	Heat-inducible transcription repressor of Class I heat shock genes, *hrcA*
Hypothetical	4.160	HP0174	JHP0160	Membrane protein, putative
Cell envelope	4.084	HP0549	JHP0496	Glutamate racemase, *glr, murl*
DNA metabolism	3.946	HP1383	N/A	Type I R-M system specificity subunit, *hsdS6*
Protein synthesis and processing	3.808	HP1497	JHP1390	Peptidyl-tRNA hydrolase, *pth*
Biosynthesis and metabolism	3.731	HP1254	JHP1175	Biotin synthesis protein, *bioC*
Motility	3.617	HP0752	JHP0689	Flagellar hook-associated protein 2 (HAP2), *fliD*
Hypothetical	3.527	HP0287	JHP0272	Hypothetical protein
Conserved hypothetical	3.424	HP0926	JHP0860	Conserved hypothetical protein
Conserved hypothetical	3.412	HP0965	JHP0899	Remnant pseudogene of an ancestral ATP/GTP-binding protein
Hypothetical	3.353	HP0754	JHP0691	Hypothetical protein

Biosynthesis and metabolism	3.285	HP0240	JHP0225	Octaprenyl-diphosphate synthase, *ispB*
Biosynthesis and metabolism	3.221	HP0211	JHP0197	Cysteine-rich protein A, *hcpA*
DNA/RNA synthesis and processing	3.217	HP0602	JHP0549	Novel 3-methyladenine DNA glycosylase involved in base excision repair, *magIII*
Hypothetical	3.159	HP1176	N/A	Hypothetical protein
Biosynthesis and metabolism	3.136	HP1461	JHP1354	Cytochrome c551 peroxidase
Motility	3.112	HP1119	JHP1047	Flagellar hook-associated protein 1 (HAP1), *flgK*
Amino acid biosynthesis	3.040	HP0107	JHP0099	Cysteine synthetase, *cysK*
Conserved hypothetical	2.825	HP1026	JHP0398	Conserved hypothetical helicase-like protein
Transporter	2.661	HP1082	JHP0343	Multidrug resistance protein, *msbA*
Hypothetical	2.631	HP0918	JHP0852	Hypothetical protein
Protein synthesis and processing	2.598	HP0223	JHP0209	DNA repair protein, *radA*
Biosynthesis of cofactors, prosthetic groups, and carriers	2.589	HP0221	JHP0207	NifU-like protein involved in [Fe-S] cluster assembly, *nifU*

* Genes listed here are according to microarray analysis differed more than 2.5-fold in *H. pylori* NTUH-S1 glutaraldehyde treatment compared to *H. pylori* NTUH-S1 without glutaraldehyde treatment.

† ORFs are according to the whole genome sequences of *H. pylori* 26695 and J99.

♯ Common name and function of each ORF are according to the PyloriGene database (http://genolist.pasteur.fr/PyloriGene).

Table S2. Genes were downregulated after glutaraldehyde treatment*

Cellular role category	Fold change	ORF HP no.†	ORF JHP no.†	Common name and function ♯
Central intermediary metabolism	10.870	HP1186	JHP1112	Carbonic anhydrase, *cah*
Transport and binding protein	7.246	HP1400	JHP1426	Iron(Ⅲ) dicitrate transport protein, *fecA3*
Fatty acid and phospholipids metabolism	6.329	HP0690	JHP0638	Acetyl coenzyme A acetyltransferase (thiolase) involved in the TCA cycle, *fadA*
Amino acid biosynthesis	5.952	HP0691	JHP0637	Succinyl-CoA-transferase subunit A of the succinyl-coenzyme A (CoA) involved in the TCA cycle, *yxjD*
Hypothetical	5.814	HP1175	JHP1102	Integral membrane protein
Amino acid biosynthesis	4.808	HP0695	JHP0633	N-methylhydantoinase
Fatty acid and phospholipids metabolism	4.587	HP0090	JHP0083	Malonyl coenzyme A-acyl carrier protein transacylase, *fabD*
Fatty acid and phospholipids metabolism	4.348	HP1045	N/A	Acetyl-CoA synthetase, *acoE*
Transport and binding protein	4.184	HP1512	JHP1405	Nickel-regulated outer membrane protein, *frpB-3*
Energy metabolism	4.016	HP0193	JHP0179	Fumarate reductase, cytochrome b subunit, *frdC*
Biosynthesis of cofactors, prosthetic groups, and carriers	3.984	HP1582	JHP1489	Pyridoxal phosphate biosynthetic protein J, *pdxJ*
Hypothetical	3.788	HP0489	JHP0441	Hypothetical protein
Central intermediary metabolism	3.717	HP0089	JHP0082	S-adenosylhomocysteine nucleosidase involved in S-adenosylmethionine recycling into methionine, *pfs*
Energy metabolism	3.559	HP0692	JHP0636	Succinyl-CoA-transferase subunit B of the succinyl-coenzyme A (CoA) involved in the TCA cycle, *yxjE*
Transport and binding protein	3.559	HP0299	JHP0284	Dipeptide ABC transporter, permease protein, *dppB*
Transport and binding protein	3.546	HP0214	JHP0200	Sodium-dependent transporter, *huNaDC-1*
Hypothetical	3.534	HP0697	JHP0631	Hypothetical protein
Cell envelope	3.390	HP0253	JHP0238	Outer membrane protein, *hopG*
Energy metabolism	3.175	HP0696	JHP0632	N-methylhydantoinase
Hypothetical	3.115	HP0015	JHP0013	Hypothetical protein
Conserved hypothetical	3.067	HP0709	JHP0648	Conserved hypothetical protein
Energy metabolism	3.040	HP0588	JHP0536	OorD subunit of the 2-oxoglutarate oxidoreductase involved in the TCA cycle, *oorD*
Amino acid biosynthesis	3.012	HP0096	JHP0088	D-2-hydroxyacid dehydrogenase

Cell envelope	2.994	HP1167	JHP1094	Outer membrane protein, *hofH*
Cell envelope	2.967	HP0710	JHP0649	Outer membrane protein, *homA*
Biosynthesis of cofactors, prosthetic groups, and carriers	2.817	HP1118	JHP1046	Gamma-glutamyltranspeptidase, *ggt*
Transport and binding protein	2.770	HP0300	JHP0285	Dipeptide ABC transporter, permease protein, *dppC*
Energy metabolism	2.762	HP0590	JHP0538	OorB subunit of the 2-oxoglutarate oxidoreductase involved in the TCA cycle, *oorB*
Transport and binding proteins	2.717	HP0693	JHP0635	Short-chain fatty acids transporter, *atoE*
Fatty acid and phospholipid metabolism	2.710	HP0891	JHP0824	Acyl coenzyme A thioesterase, *vdlD*
Conserved hypothetical	2.646	HP0920	JHP0854	Integral membrane protein

* Genes listed here are according to microarray analysis differed more than 2.5-fold downregulated in *H. pylori* NTUH-S1 glutaraldehyde treatment compared to *H. pylori* NTUH-S1 without glutaraldehyde treatment.

† ORFs are according to the whole genome sequences of *H. pylori* 26695 and J99.

♯ Common name and function of each gene are according to the PyloriGene database (http://genolist.pasteur.fr/PyloriGene).
